# Hyperglycemia in non‐obese patients with type 2 diabetes is associated with low muscle mass: The Multicenter Study for Clarifying Evidence for Sarcopenia in Patients with Diabetes Mellitus

**DOI:** 10.1111/jdi.13070

**Published:** 2019-06-01

**Authors:** Ken Sugimoto, Yasuharu Tabara, Hiroshi Ikegami, Yasunori Takata, Kei Kamide, Tome Ikezoe, Eri Kiyoshige, Yukako Makutani, Hiroshi Onuma, Yasuyuki Gondo, Kazunori Ikebe, Noriaki Ichihashi, Tadao Tsuboyama, Fumihiko Matsuda, Katsuhiko Kohara, Mai Kabayama, Masahiro Fukuda, Tomohiro Katsuya, Haruhiko Osawa, Yoshihisa Hiromine, Hiromi Rakugi

**Affiliations:** ^1^ Department of Geriatric and General Medicine Osaka University Graduate School of Medicine Suita Japan; ^2^ Center for Genomic Medicine Kyoto University Graduate School of Medicine Kyoto Japan; ^3^ Department of Endocrinology, Metabolism and Diabetes Kindai University Osaka‐sayama Japan; ^4^ Department of Diabetes and Molecular Genetics Ehime University Graduate School of Medicine Toon Japan; ^5^ Department of Health Promotion Sciences Division of Health Sciences Osaka University Graduate School of Medicine Suita Japan; ^6^ Department of Physical Therapy Human Health Sciences Kyoto University Graduate School of Medicine Kyoto Japan; ^7^ Diabetes/Metabolic Endocrinology Yachiyo Medical Center Tokyo Women's Medical University Yachiyo Japan; ^8^ Department of Clinical Thanatology and Geriatric Behavioral Science Osaka University Graduate School of Human Sciences Japan; ^9^ Department of Prosthodontics, Gerodontology and Oral Rehabilitation Osaka University Graduate School of Dentistry Suita Japan; ^10^ School of Health Sciences Bukkyo University Kyoto Japan; ^11^ Department of Regional Resource Management Faculty of Collaborative Regional Innovation Ehime University Matsuyama Japan; ^12^ Fukuda Clinic Osaka Japan; ^13^ Katsuya Clinic Amagasaki Japan; ^14^ Department of Clinical Gene Therapy Osaka University Graduate School of Medicine Suita Japan

**Keywords:** Sarcopenia, Skeletal muscle mass, Type 2 diabetes

## Abstract

**Aims/Introduction:**

Hyperglycemia is a risk factor for sarcopenia when comparing individuals with and without diabetes. However, no studies have investigated whether the findings could be extrapolated to patients with diabetes with relatively higher glycemic levels. Here, we aimed to clarify whether glycemic control was associated with sarcopenia in patients with type 2 diabetes.

**Materials and Methods:**

Study participants consisted of patients with type 2 diabetes (*n* = 746, the average age was 69.9 years) and an older general population (*n* = 2,067, the average age was 68.2 years). Sarcopenia was defined as weak grip strength or slow usual gait speed and low skeletal mass index.

**Results:**

Among patients with type 2 diabetes, 52 were diagnosed as having sarcopenia. The frequency of sarcopenia increased linearly with glycated hemoglobin (HbA1c) level, particularly in lean individuals (HbA1c <6.5%, 7.0%, ≥6.5% and <7.0%: 18.5%; HbA1c ≥7.0% and <8.0%: 20.3%; HbA1c ≥8.0%: 26.7%). The linear association was independent of major covariates, including anthropometric factors and duration of diabetes (HbA1c <6.5%: reference; ≥6.5% and <7.0%: odds ratio [OR] 4.38, *P *=* *0.030; HbA1c ≥7.0% and <8.0%: 4.29, *P *=* *0.024; HbA1c ≥8.0%: 7.82, *P *=* *0.003). HbA1c level was specifically associated with low skeletal mass index (HbA1c ≥8.0%: OR 5.42, *P *<* *0.001) rather than weak grip strength (OR 1.89, *P *=* *0.058) or slow gait speed (OR 1.13, *P *=* *0.672). No significant association was observed in the general population with a better glycemic profile.

**Conclusions:**

Poor glycemic control in patients with diabetes was associated with low muscle mass.

## Introduction

Sarcopenia is a composite phenotype defined by a combination of excessive loss of muscle mass, weakening of muscle strength and decline of physical function^1^. Multiple factors, including old age, immobility, malnutrition, neurodegeneration and chronic inflammation, have been suggested to be associated with the development of sarcopenia^2^; however, the most important risk factors in this era, when frequency of obesity is rapidly increasing worldwide, might be insulin resistance and type 2 diabetes. Indeed, individuals with type 2 diabetes have weaker muscle strength and quality^3^, ^4^, ^5^, ^6^ compared with non‐diabetic control individuals. Even in East Asian populations, in which individuals have a relatively smaller body size compared with that of individuals in Western countries, the frequency of sarcopenia, estimated to be 6–12% in general populations^7^, ^8^, was high in type 2 diabetes patients in a case–control analysis^9^; it was also high in a cross‐sectional analysis in an older population^10^. In addition to the cross‐sectional relationship, type 2 diabetes is a risk factor for the longitudinal decline in lower extremity muscle mass^11^ and strength^11^, ^12^ in older adults. Furthermore, hyperglycemia is associated with deterioration of physical performance^13^.

However, as the majority of previous studies on sarcopenia used a cross‐sectional setting by comparing patients with diabetes and non‐diabetic controls, it is unclear whether sarcopenia worsens in relation to the level of glycemic control in patients with diabetes. If glycemic control levels were identified as a risk factor for sarcopenia among patients with diabetes, the findings might be useful in diabetes care, as it will clarify the clinical importance of glycemic control in the prevention of not only end‐organ damage, but also sarcopenia and frailty in old age. Furthermore, as lower physical performance in patients with diabetes is associated with cardiovascular morbidities^14^, ^15^, total mortality^15^ and hospitalization^16^, it also should be clarified whether hyperglycemia is associated with lower physical performance.

Here, we carried out a multicenter cross‐sectional study to clarify the association of glycemic control levels with sarcopenia, as well as its components (namely, muscle mass assessed using the skeletal muscle index [SMI], a measure of lean body mass extremity, muscle strength and physical performance) in patients with type 2 diabetes who were regularly treated by physicians. In addition, the results of the cross‐sectional association analysis in independent general populations were considered to show the relationship between better glycemic profiles and sarcopenia.

## Methods

### Patients with diabetes

The present study analyzed a dataset of the ongoing Multicenter Study for Clarifying Evidence for Sarcopenia in Patients with Diabetes Mellitus (the MUSCLES‐DM study), currently including a total of 768 independent ambulatory patients with stable type 2 diabetes aged ≥40 years at recruitment. Participants were recruited between May 2016 and December 2017 from patients receiving treatment and regularly visiting three university hospitals (Osaka University, Kindai University and Ehime University) and two general clinics (Fukuda Clinic and Katsuya Clinic). Among them, this study analyzed 746 patients who finished a physical performance test required for diagnosis of sarcopenia and whose clinical data, including disease histories, treatment regimens and plasma levels of glycemic parameters, were available for this study. We used glycated hemoglobin (HbA1c), but not other glycemic parameters, namely plasma glucose and insulin levels, as an index representing insulin resistance, insulin secretion and therapeutic effect, because all study patients were taking oral antidiabetic medications or insulin therapy. Exercise habits (at least twice a week for 30 min for over a year)^17^ were investigated using a structured questionnaire.

All study procedures were approved by the ethics committee of the three universities, and written informed consent was obtained from all participants.

### Community residents and older adult populations

We analyzed the following two datasets to compare results of the analysis of patients with type 2 diabetes with: (i) an older subpopulation (aged ≥60 years) of the Nagahama study (*n* = 2,067), which consisted of general community residents; and (ii) the Septuagenarians, Octogenarians, Nonagenarians Investigation with Centenarians (SONIC) study (*n* = 559) including randomly selected older adults aged 69–71 years (SONIC 70) or 79–81 years (SONIC 80) during the baseline investigation. Detailed characteristics, as well as methods of sarcopenia assessment of these populations, are described in Data S1.

### Assessment of sarcopenia

Sarcopenia was defined using a modified definition of the Asian Working Group for Sarcopenia^1^: weak handgrip (<26 kg for men, <18 kg for women) or slow usual gait speed (<1.0 m/s) and low SMI (<7.0 kg/m^2^ for men, <5.7 kg/m^2^ for women). We adopted <1.0 m/s instead of <0.8 m/s as a cut‐off point of slow gait speed according to the Japanese version of the Cardiovascular Health Study criteria^18^.

### Measurement of grip strength

Grip strength of the dominant hand was measured using a standard digital grip dynamometer (Grip‐D; Takei Scientific Instruments Co., Ltd., Niigata, Japan). Measurements were taken twice in a sitting position with the arm positioned horizontal to the ground. The participants were instructed to adjust the handle of the dynamometer so that it would be under the second phalanx when gripped. The mean values of all measurements were used for analysis.

### Measurement of usual gait speed

Usual gait speed measurement was carried out using a 2.44‐m (Katsuya Clinic) or 4‐m (other hospitals) walkway with a 1‐m approach way. Measurements were obtained by an accompanying person using a digital timer. Measurements were taken twice, and the average value was used for the analysis. The gait speeds measured using the different walking distances were adjusted using equations (Figure S1) to avoid potential misclassification.

### Assessment of SMI

The appendicular lean mass and fat mass was estimated using bioelectrical impedance analysis devices (MC‐780A; Tanita Co., Tokyo, Japan). The validity and reproducibility of appendicular lean mass and fat mass measurements by the segmental multiple‐frequency bioelectrical impedance analysis were reported to be comparable to dual‐energy X‐ray absorptiometry^19^, ^20^, ^21^, ^22^ and hydrostatic weighing^19^. SMI was obtained by dividing appendicular lean mass by squared body height^21^.

### Statistical analysis

**Figure 1:**
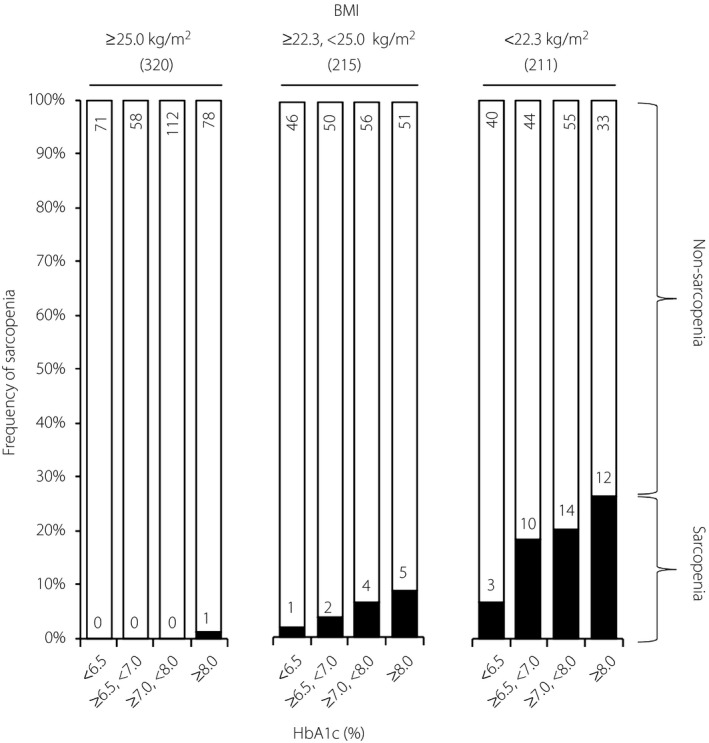
Association between glycated hemoglobin (HbA1c) levels and sarcopenia in patients with type 2 diabetes. The frequency of sarcopenia by body mass index and plasma HbA1c levels. Study participants were subdivided at a body mass index (BMI) of 25 kg/m^2^, a criterion of obesity in Japan, and 23.3 kg/m^2^, which corresponds to the median among the remaining non‐obese participants. The numbers of participants in each BMI subgroup are shown in the columns and parentheses.

Calculated values were presented as the mean ± standard deviation of frequency. Group differences in numeric variables were assessed using analysis of variance, whereas frequency differences were assessed using a χ^2^‐test. The association of HbA1c levels with sarcopenia indices (Table 2; Figure 1b) was analyzed using logistic regression analysis including stratified HbA1c levels as dummy variables; the lowest HbA1c subgroup (≤6.5%) was considered as a reference. All statistical analyses were carried out using JMP statistical software (JMP Pro version 13.2.0; SAS Institute, Cary, NC, USA). A *P*‐value <0.05 was considered statistically significant.

## Results

The clinical characteristics of the study participants (type 2 diabetes) are summarized in Table S1. The mean age was 69.9 ± 9.1 years, and the age range was 38–96 years, while the duration of diabetes ranged 0–60 years. Sex differences (men 60.3%) in the clinical characteristics are shown in Table S2.

Among the study participants, 52 individuals (7.0%) were diagnosed as having sarcopenia (Table 1). When the participants were subdivided by HbA1c level, the frequency of sarcopenia linearly increased in accordance with HbA1c levels (HbA1c <6.5%: 2.5%, HbA1c ≥6.5% and <7.0%: 7.3%, HbA1c ≥7.0%, HbA1c <8.0%: 7.5% and HbA1c ≥8.0%: 10.0%, *P *=* *0.053). This linear association was more prominent when the participants were stratified by BMI level at 25 kg/m^2^, a criterion of obesity in Japan, and 23.3 kg/m^2^, which corresponds to the median among the remaining non‐obese participants (Figure 1), due to the large differences in BMI between sarcopenia and non‐sarcopenia groups (Table 1).

**Table 1:** Clinical characteristics of patients with type 2 diabetes with and without sarcopenia

	Sarcopenia (52)	Non‐sarcopenia (694)	*P*
Age (years)	73.8 ± 6.9	69.6 ± 9.2	0.001
Male (%)	61.5	60.2	0.853
BMI (kg/m^2^)	20.7 ± 2.3	25.0 ± 4.1	<0.001
Fat mass (kg)	14.0 ± 5.2	18.4 ± 8.5	<0.001
Duration of diabetes (years)†	15.8 ± 11.6	15.7 ± 10.1	0.899
Duration of treatment of diabetes (years)‡	14.1 ± 10.3	14.1 ± 9.5	0.997
Exercise habit (%)	42.3	55.2	0.072
Cardiovascular diseases (%)	42.3	34.2	0.233
Retinopathy, PDR or post‐PC (%)§	12.2	12.7	0.925
Nephropathy, stage ≥3 (%)¶	12.8	8.3	0.295
Medication			
Hypertension (%)	59.6	68.7	0.174
Dyslipidemia (%)	59.6	56.3	0.646
Hyperglycemia			
Sulfonylureas (%)	17.3	27.5	0.109
Glinides (%)	15.4	8.5	0.094
Biguanides (%)	25.0	37.8	0.066
Thiazolidinediones (%)	11.5	15.0	0.499
DPP‐4 inhibitors (%)	59.6	60.2	0.930
GLP‐1 analogs (%)	5.8	5.2	0.856
SGLT‐2 inhibitors (%)	5.8	13.8	0.098
Insulin (%)	59.6	71.8	0.063
Plasma markers			
Albumin (mg/dL)	4.1 ± 0.4	4.2 ± 0.4	0.011
Creatinine (mg/dL)	1.0 ± 0.9	0.9 ± 0.3	0.025
HbA1c (%)	7.7 ± 1.3	7.4 ± 1.3	0.047
Sarcopenia			
Skeletal mass index (kg/m^2^)	6.1 ± 0.6	7.6 ± 1.2	<0.001
Low skeletal mass index (%)	100.0	7.1	<0.001
Grip strength (kg)	20.3 ± 6.3	28.9 ± 9.3	<0.001
Weak grip strength (%)	73.1	17.6	<0.001
Usual gait speed (m/s)	1.02 ± 0.28	1.18 ± 0.26	<0.001
Slow usual gait speed (%)	63.5	22.9	<0.001
Arm muscle quality	5.8 ± 1.5	6.5 ± 1.6	0.001

Values are shown as the mean ± standard deviation or frequency. Statistical significance was assessed by analysis of variance or a χ^2^‐test. Sarcopenia was defined as weak grip strength (<26 kg for men, <18 kg for women) or slow usual gait speed (<1.0 m/s) and low skeletal mass index (<7.0 kg/m^2^ for men, <5.7 kg/m^2^ for women). Arm muscle quality was calculated by dividing grip strength by arm muscle mass. Cardiovascular diseases include cerebrovascular disease, ischemic heart disease and peripheral artery disease. Data are available for ^†^729, ^‡^698, ^§^709 and ^¶^684 patients. BMI, body mass index; DPP‐4, dipeptidyl peptidase‐4; GLP‐1, glucagon‐like peptide‐1; HbA1c, glycated hemoglobin; PC, photocoagulation; PDR, proliferative diabetic retinopathy; SGLT‐2, sodium–glucose cotransporter 2.

As other factors also differed significantly between sarcopenia and non‐sarcopenia groups (Table 1), logistic regression analyses were carried out to clarify whether the positive association between HbA1c level and sarcopenia was independent of these covariates. The results showed that the positive association of HbA1c level (Table 1, model 1) was independent of the basic covariates (model 2), as well as of the duration of diabetes and existence of retinopathy (model 3), even when HbA1c level was included as a continuous variable in model 1 (odds ratio [OR] 1.29, 95% confidence interval [CI] 1.01–1.74; *P *=* *0.041). Furthermore, when fat mass was included in the model, it was identified as a positive determinant for sarcopenia (OR 1.54, 95% CI 1.34–1.76) independently of BMI, for which it showed an inverse association (OR 0.29, 95% CI 0.21–0.39). In this model, the association of HbA1c level with sarcopenia remained statistically significant (HbA1c ≥6.5% and <7.0%: OR 4.70, *P *=* *0.042; HbA1c ≥7.0% and <8.0%: OR 5.11, *P *=* *0.024; HbA1c ≥8.0%: OR 10.87, *P *=* *0.002).

In the subanalysis of patients with a BMI <23.3 kg/m^2^ (Table 2, model 4), the association of HbA1c level remained significant with similar ORs, suggesting an additive effect of BMI in the association between HbA1c level and sarcopenia. The interaction term between HbA1c level and BMI was indeed insignificant when it was added to regression model 1 (*P *=* *0.413). The complete results of this regression analysis are shown in Table S3.

**Table 2:** Logistic regression analysis for sarcopenia

Model	*n*	Adjusted factors	HbA1c (%)						
			<6.5	≥6.5 and <7.0		≥7.0 and <8.0		≥8.0	
				OR (95% CI)	*P*	OR (95% CI)	*P*	OR (95% CI)	*P*
Model 1	746	None	Reference	3.10 (0.98–9.82)	0.055	3.17 (1.05–9.54)	0.040	4.36 (1.44–13.17)	0.009
Model 2	746	Basic factors	Reference	4.54 (1.20–17.15)	0.025	4.77 (1.36–16.80)	0.015	7.20 (1.94–26.67)	0.003
Model 3	642	Fully adjusted	Reference	4.30 (1.11–16.65)	0.035	4.48 (1.24–16.17)	0.022	7.65 (1.95–30.00)	0.003
Model 4	211 (BMI <22.3 kg/m^2^)	Basic factors	Reference	6.32 (1.32–30.34)	0.021	5.38 (1.25–23.05)	0.023	7.55 (1.59–35.79)	0.011

Sarcopenia was defined as weak grip strength (<26 kg for men, <18 kg for women) or slow usual gait speed (<1.0 m/s) and low skeletal mass index (<7.0 kg/m^2^ for men, <5.7 kg/m^2^ for women). Adjusted factors were as follows: basic factors were age, sex, body mass index, exercise habit, serum albumin, oral antihyperglycemic drugs, insulin therapy and cardiovascular diseases (ischemic heart diseases, cerebrovascular diseases and peripheral artery diseases); fully adjusted were basic factors plus duration of diabetes and retinopathy. Full results of the logistic regression analyses are shown in Table S3. BMI, body mass index; CI, confidence interval; HbA1c, glycated hemoglobin; OR, odds ratio.

In a separate analysis for the three components of sarcopenia (Table 3; Table S4), HbA1c level was specifically associated with low SMI, while clear associations were not observed with grip strength or slow gait speed.

**Table 3:** Logistic regression analysis for sarcopenia indices

	HbA1c (%)						
	<6.5	≥6.5 and <7.0		≥7.0 and <8.0		≥8.0	
		OR (95% CI)	*P*	OR (95% CI)	*P*	OR (95% CI)	*P*
Low skeletal muscle index	Reference	3.29 (1.31–8.23)	0.011	2.61 (1.09–6.24)	0.030	5.35 (2.07–13.86)	<0.001
Weak grip strength	Reference	1.07 (0.55–2.07)	0.834	1.46 (0.80–2.67)	0.221	1.95 (1.00–3.80)	0.050
Slow gait speed	Reference	0.66 (0.37–1.16)	0.153	1.00 (0.60–1.65)	0.994	1.16 (0.65–2.06)	0.612

Total *n* = 746. Low skeletal mass index: <7.0 kg/m^2^ for men, <5.7 kg/m^2^ for women; weak grip strength: <26 kg for men, <18 kg for women; slow usual gait speed: <1.0 m/s. Adjusted factors were age, sex, body mass index, exercise habit, serum albumin, oral antihyperglycemic drugs, insulin therapy and cardiovascular diseases (ischemic heart diseases, cerebrovascular diseases and peripheral artery diseases). Full results of the logistic regression analyses are shown in Table S3. HbA1c, glycated hemoglobin; CI, confidence interval; OR, odds ratio.

The clinical characteristics of the study populations from the Nagahama study and SONIC study are summarized in Tables S5 and S6, respectively. The frequencies of sarcopenia in these populations by HbA1c and BMI levels are shown in Figures S2 and S3. Although sarcopenia was also frequently observed in the lower BMI subgroups in these populations, the frequency differences in sarcopenia among the HbA1c subgroups were modest compared with those observed in regularly treated patients with type 2 diabetes.

**Figure 2:**
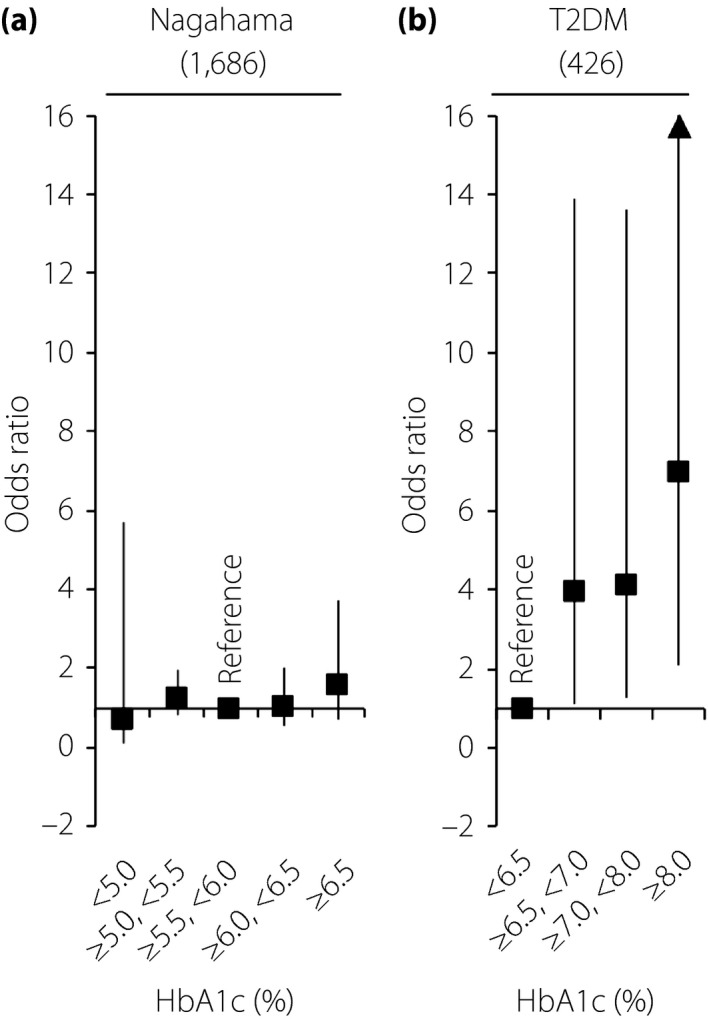
Adjusted odds ratio for sarcopenia in non‐obese patients. (a) General population (the Nagahama study). (b) Patients with type 2 diabetes. Patients whose body mass index was >25 kg/m^2^ were excluded from the analysis. The odds ratio adjusted for age, sex and body mass index was calculated using the (a) intermediate or (b) lowest glycated hemoglobin (HbA1c) subgroup as a reference. T2DM, type 2 diabetes.

Figure 2 shows age, sex and BMI‐adjusted ORs of the HbA1c subgroups for sarcopenia in non‐obese (BMI <25.0 kg/m^2^) individuals. In contrast to the Nagahama (Figure 2a) and SONIC (Figure S4) populations with relatively lower HbA1c levels, patients with type 2 diabetes (Figure 2b) and poor glycemic control showed a significantly higher OR for sarcopenia.

## Discussion

In the present cross‐sectional study of patients with type 2 diabetes, glycemic control levels that were assessed using HbA1c levels were independently associated with sarcopenia, particularly in non‐obese individuals. This association was more prominent in the analysis with muscle mass rather than with muscle performance, namely grip strength and gait speed.

HbA1c was not associated with sarcopenia in the Nagahama population with near‐normal HbA1c levels, suggesting that chronic hyperglycemia in patients with type 2 diabetes should be considered as a risk factor for muscle mass decline. Although the frequency of sarcopenia did not differ substantially between the diabetes patients and the Nagahama population in the analysis of the total population, the twofold higher frequency of sarcopenia in the lean diabetes patients showed that the deleterious effect of hyperglycemia depends on the patient's bodyweight.

The association between hyperglycemia and sarcopenia might be bidirectional. Although hyperglycemia could contribute to the longitudinal decline in muscle mass^11^ and strength^11^, ^12^, reduced glucose disposal capacity due to loss of muscle mass and quality might further deteriorate glucose metabolism^23^, ^24^. The current study showed a lack of association between HbA1c levels and sarcopenia in the general population and among older adults in whom SMI was relatively lower than that in patients with type 2 diabetes; this suggests a causal role of hyperglycemia for lowering muscle mass in patients with type 2 diabetes. Although a detailed mechanism for the deleterious effects of hyperglycemia has not been fully elucidated, accumulation of advanced glycation end‐products^25^, ^26^, as well as changes in skeletal muscle extracellular matrix, might be possible reasons^27^. In addition to hyperglycemia per se, impaired insulin action might also be an underlying factor that could explain the relationship between hyperglycemia and sarcopenia, because insulin is a well‐known anabolic hormone that contributes to muscle protein synthesis.

In patients with type 2 diabetes, the frequency of sarcopenia was linearly increased with HbA1c levels, and the association was independent of possible covariates. Because no similar association was observed in a general population and in an older adult population with near‐normal HbA1c levels, an HbA1c level of 6.5% might be a considerable threshold value (Figure 2) when sarcopenia prevention is considered as a primary purpose. In contrast, most guidelines for treatment of type 2 diabetes^28^, ^29^ usually recommended higher HbA1c levels as a target value based on findings regarding micro‐ and macrovascular complications of diabetes. Because the present study was cross‐sectional, further longitudinal observational studies, as well as prospective intervention studies with a certain HbA1c level as a glycemic target, are required to clarify an optimal glycemic control level for the prevention of sarcopenia.

Previous cross‐sectional studies that reported a harmful effect of diabetes on sarcopenia^3^, ^4^, ^5^, ^6^, ^9^, ^10^ were based on findings that the frequency of sarcopenia was significantly higher in patients with diabetes than in non‐diabetic control participants. However, no studies have reported whether the frequency of sarcopenia was increased by exacerbation of glycemic control in patients with diabetes. Therefore, a novel contribution of the present study is that it clarifies a linear relationship between hyperglycemia, as assessed by HbA1c levels, and the frequency of sarcopenia in patients with diabetes. The present results further clarified that the harmful effect of hyperglycemia was specific to SMI and not associated with physical performance, namely grip strength and gait speed, as well as muscle quality; in contrast, previous studies found lower physical performance^3^, ^4^, ^5^, ^6^ and physical functional decline^13^ in patients with diabetes in comparison with non‐diabetic controls. A reason for the discrepancy might be the different study setting; that is, previous studies found lower physical performance in patients with relatively mild diabetes, suggesting that functional decline in physical performance might precede muscle mass decline. Furthermore, as patients with type 2 diabetes usually have a larger body size than non‐diabetic controls, the weight load on extremities might prevent the loss of muscle mass and consequently weaken the pathophysiological relationship between diabetes and muscle mass decline during the early phase of diabetes.

It has been reported that decreases in muscle mass and strength might depend on the duration of diabetes^30^, although the results of our regression analysis showed a modest inverse association with disease duration. Certain types of drugs, namely, insulin sensitizers and dipeptidyl peptidase‐4 inhibitors, attenuate the decline of muscle mass and muscle function^31^, ^32^. As the frequencies of patients who were prescribed insulin sensitizers and dipeptidyl peptidase‐4 inhibitors were approximately 40 and 60% in our study population, respectively, the short time‐lag after diagnosis to treatment of diabetes and use of antihyperglycemic drugs with a possible antisarcopenic activity might help explain the inverse association between duration of diabetes and sarcopenia in our study population.

The association between HbA1c level and sarcopenia was independent of history of cardiovascular disease and retinopathy (Table 3, model 3), which influence the diagnosis of sarcopenia by affecting physical performance, particularly gait speed. The association of HbA1c level was also independent of antihyperglycemic therapy, although certain drugs, such as insulin sensitizers and dipeptidyl peptidase‐4 inhibitors^31^, ^32^, as well as sodium–glucose cotransporter 2 inhibitor^33^, might benefit muscle strength. The present findings might, therefore, be less biased by these potential confounding factors.

There were several limitations that warrant mention. First, we did not evaluate the involvement of insulin resistance, a possible mechanism relating diabetes and sarcopenia^34^, due to the small number of patients with type 2 diabetes who were not receiving insulin therapy and whose fasting blood samples were available (*n* = 93). Given that the frequency of taking biguanides or insulin therapy was slightly higher in the non‐sarcopenia group, further studies with detailed assessment of insulin resistance, such as the use of an oral glucose tolerance test, might help clarify this issue. Second, the number of patients with diabetes who were diagnosed with sarcopenia was limited. The harmful effects of hyperglycemia on sarcopenia, particularly in obese individuals, would be clarified by expanding the study population. Third, we did not directly measure muscle quality, such as with imaging analysis using ultrasonography or computed tomography. A detailed evaluation of muscle quality might further clarify the harmful effects of diabetes on physical performance. Fourth, daily physical activity and nutritional status, especially protein intake, which are possible modulators of the relationship between diabetes and sarcopenia, were not considered in the present study. However, we used data regarding exercise habits, and there were no frequency differences in daily exercise habits between sarcopenic patients and non‐sarcopenic controls (Table 1). Fifth, the parameters for diabetic neuropathy, which might be related to development of sarcopenia, were not available in the present study, because there have been no established quantitative parameters for diabetic neuropathy. This issue deserves further investigations. Sixth, although we did not find any association between low HbA1c levels and sarcopenia in both the Nagahama and SONIC populations, this issue also deserves further investigation. Several observational studies in older adults^35^ and in patients with diabetes^36^ reported a U‐shaped association of HbA1c levels with mortality^36^ and the incidence of frailty^35^. The results of the present study might have insufficient statistical power to investigate this issue because of the small number of participants with low HbA1c. Seventh, the present study population was Japanese. Because Asians are more likely to develop type 2 diabetes at lower BMI, the present findings might not extrapolate to other populations.

In summary, glycemic control levels in Japanese patients with type 2 diabetes were significantly associated with sarcopenia independent of possible covariates. Poorly controlled diabetes in older patients with a smaller body size requires careful attention for the prevention of sarcopenia.

## Disclosure

The authors declare no conflict of interest.

## Supporting information


**Figure S1** | Different associations between walkway distance and usual gait speed by the point of gait speed calculation.

**Figure S2** | Association between glycated hemoglobin (HbA1c) levels and sarcopenia in a general population (the Nagahama study).

**Figure S3** | Association between glycated hemoglobin (HbA1c) levels and sarcopenia in an older adult population (the Septuagenarians, Octogenarians, Nonagenarians Investigation with Centenarians [SONIC] study).

**Figure S4** | Adjusted odds ratio for sarcopenia in non‐obese individuals (the SONIC study).

**Table S1** | Clinical characteristics of patients with diabetes.

**Table S2** | Sex differences in clinical characteristics of the study patients.

**Table S3** | Full results of regression analysis for sarcopenia.

**Table S4** | Full results of regression analysis for sarcopenia indices.

**Table S5** | Clinical characteristics of the Nagahama study population.

**Table S6** | Clinical characteristics of the Septuagenarians, Octogenarians, Nonagenarians Investigation with Centenarians study population.

**Data S1** | Supplementary methods.
