# Supplementary figures and images for: Hyperglycemia in non‐obese patients with type 2 diabetes is associated with low muscle mass: The Multicenter Study for Clarifying Evidence for Sarcopenia in Patients with Diabetes Mellitus
Source: J Diabetes Investig. 2019 Jun 1;10(6):1471–9. doi: 10.1111/jdi.13070 (PMC6825926; doi:10.1111/jdi.13070)

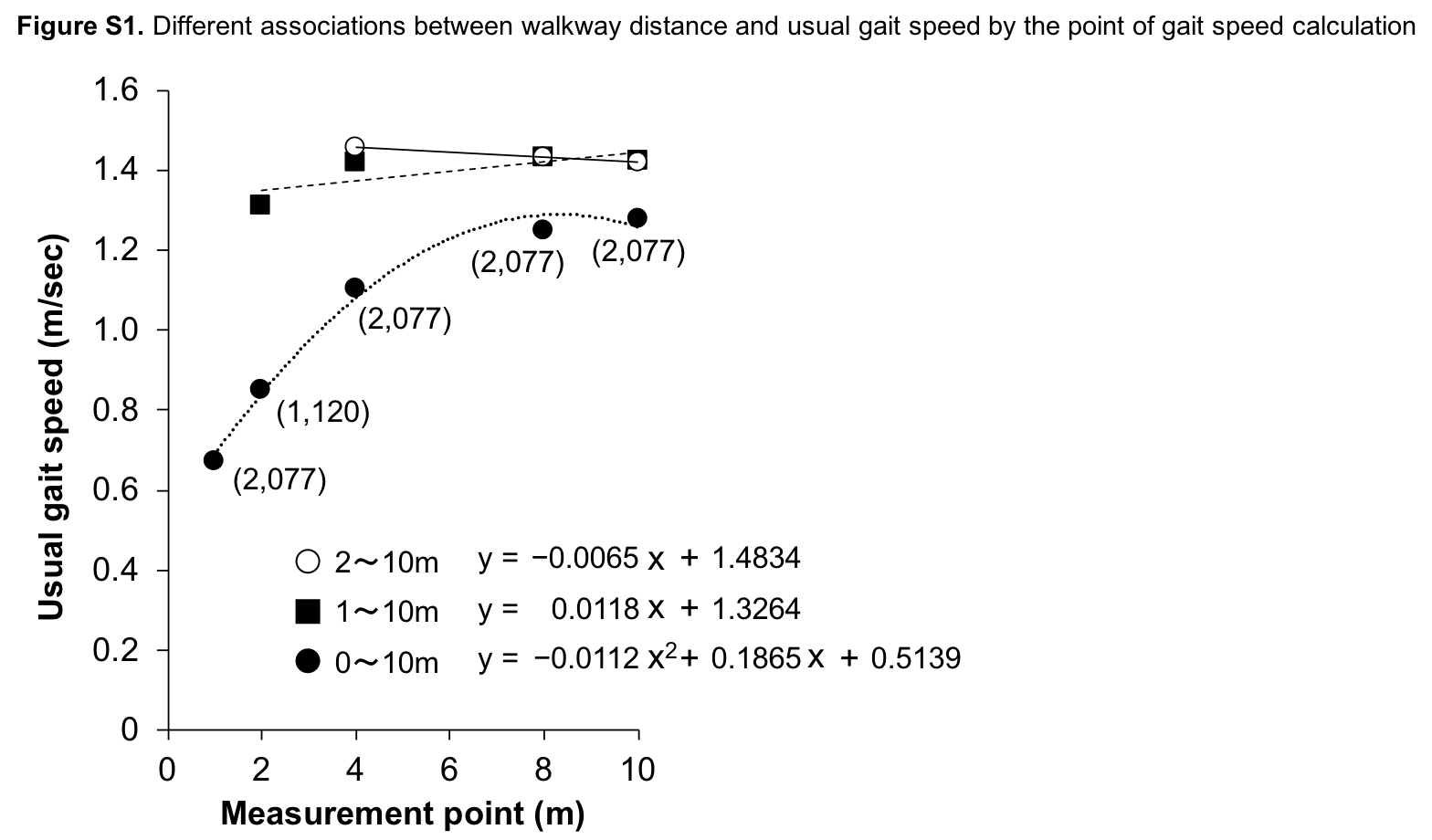

Supplement: Supplementary file 1 — Figure S1 | Different associations between walkway distance and usual gait speed by the point of gait speed calculation. [file JDI-10-1471-s001.tiff]

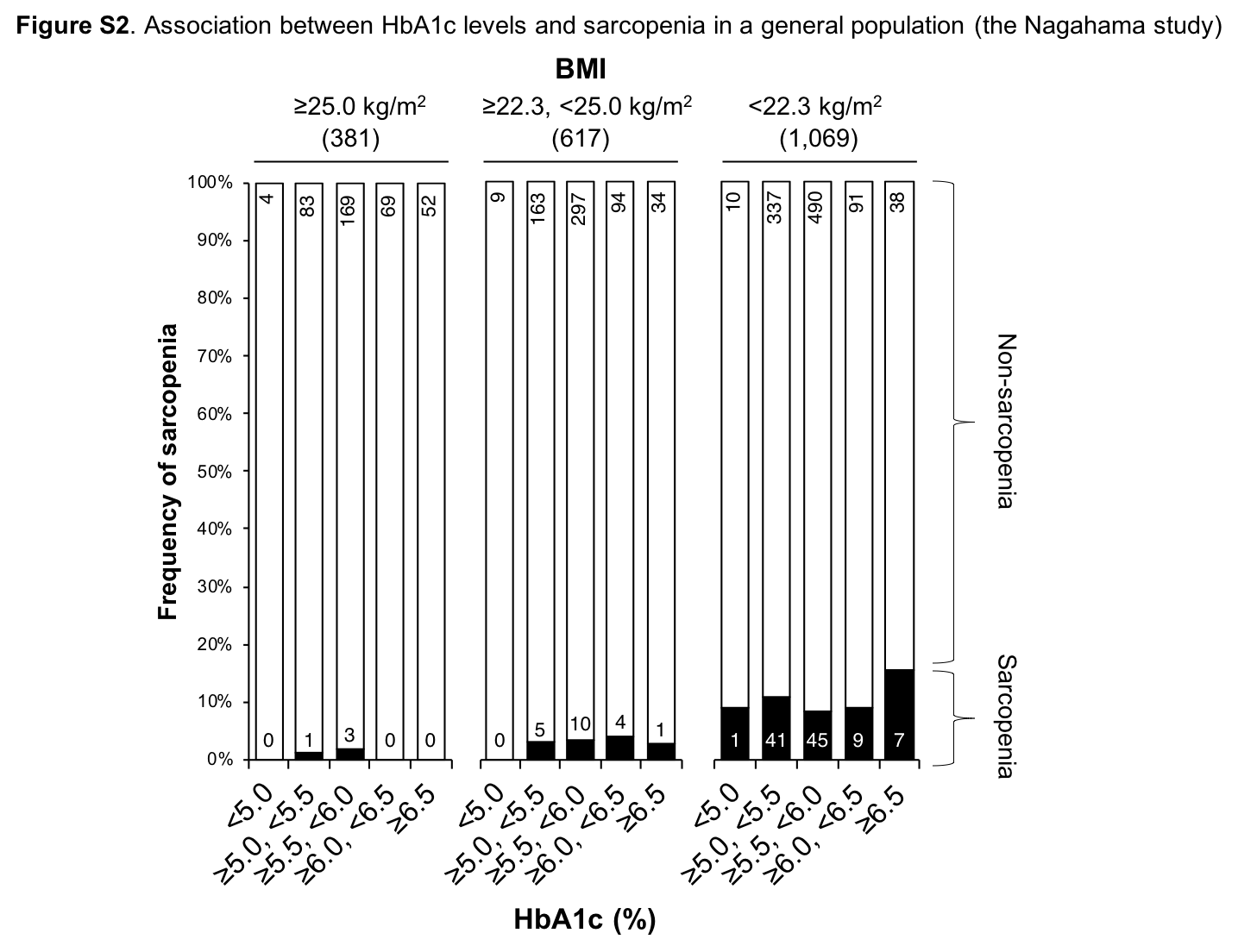

Supplement: Supplementary file 2 — Figure S2 | Association between glycated hemoglobin (HbA1c) levels and sarcopenia in a general population (the Nagahama study). [file JDI-10-1471-s002.tiff]

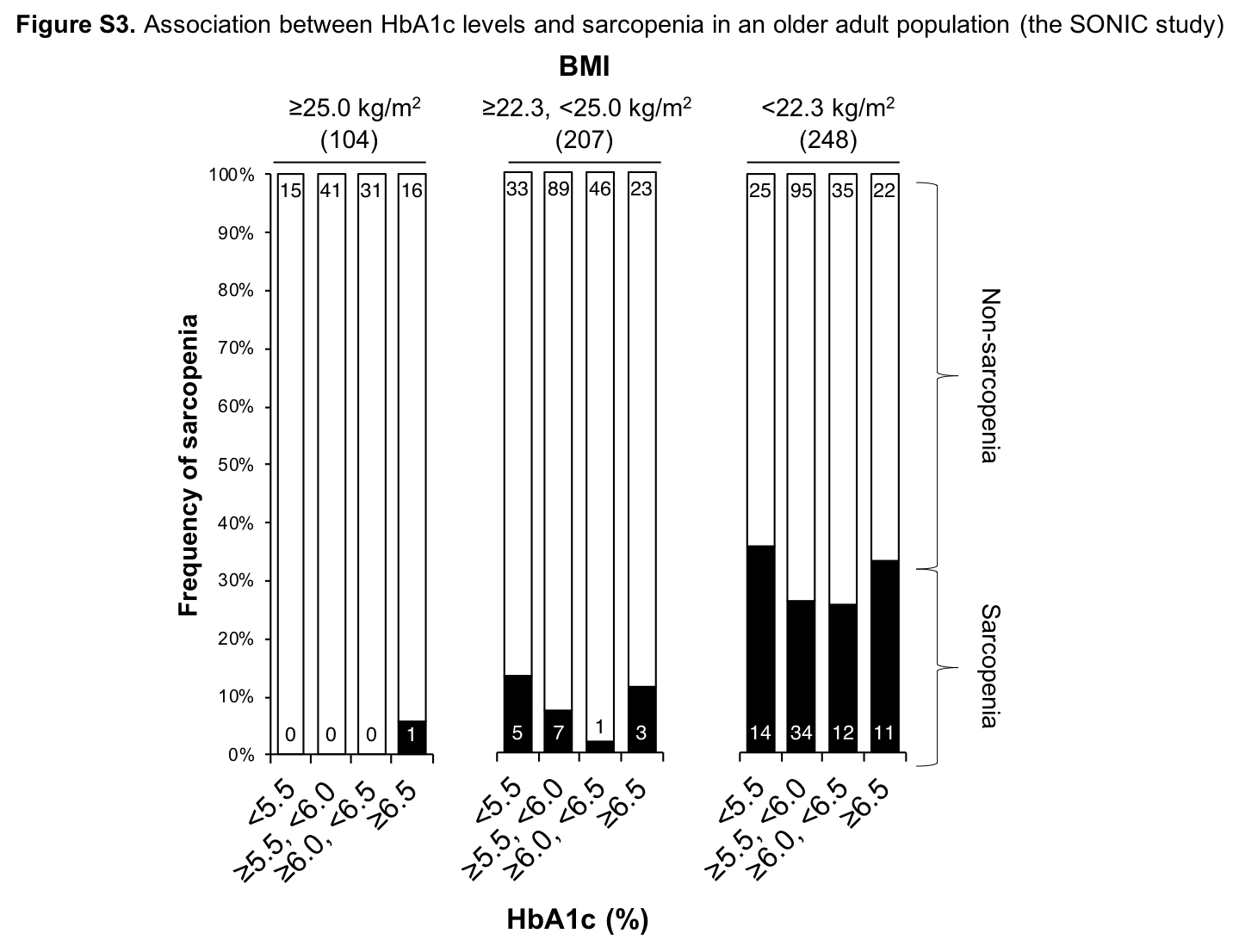

Supplement: Supplementary file 3 — Figure S3 | Association between glycated hemoglobin (HbA1c) levels and sarcopenia in an older adult population (the Septuagenarians, Octogenarians, Nonagenarians Investigation with Centenarians [SONIC] study). [file JDI-10-1471-s003.tiff]

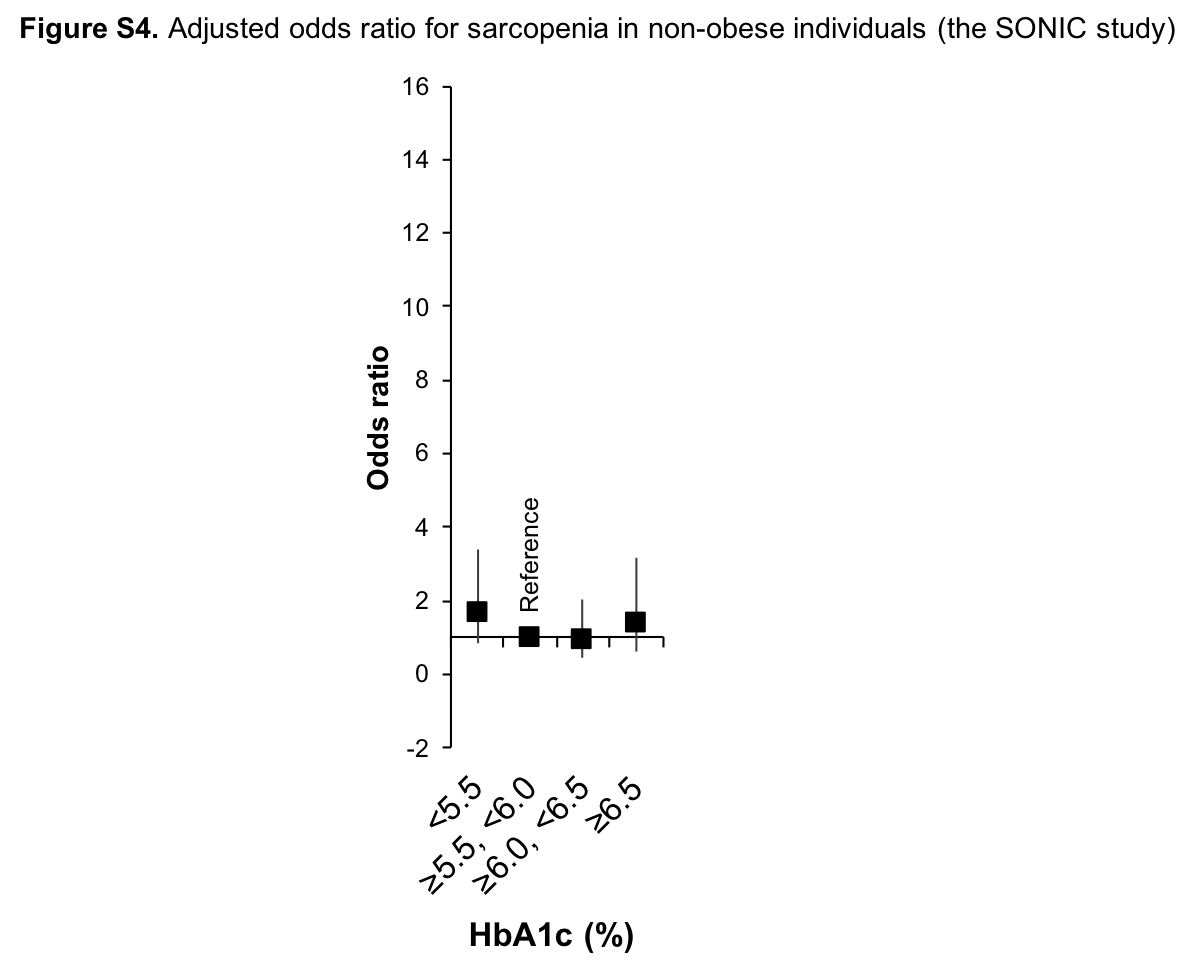

Supplement: Supplementary file 4 — Figure S4 | Adjusted odds ratio for sarcopenia in non‐obese individuals (the SONIC study). [file JDI-10-1471-s004.tiff]
