# Supplementary material for: Hyperglycemia in non‐obese patients with type 2 diabetes is associated with low muscle mass: The Multicenter Study for Clarifying Evidence for Sarcopenia in Patients with Diabetes Mellitus
Source: J Diabetes Investig. 2019 Jun 1;10(6):1471–9. doi: 10.1111/jdi.13070 (PMC6825926; doi:10.1111/jdi.13070)
Supplement: Supplementary file 5 — Table S1 | Clinical characteristics of patients with diabetes. [file JDI-10-1471-s005.docx]

**Supplementary table 1.** Clinical characteristics of patients with diabetes (n=746)

| Age (years) | | 69.9±9.1 |
| --- | --- | --- |
| Sex (male, %) | | 60.3 |
| BMI (kg/m^2^) | | 24.7±4.1 |
| Fat mass (kg) | | 18.1±8.4 |
| Duration of diabetes (years) ^†^ | | 15.7±10.2 |
| Duration of treatment of diabetes (years) ^‡^ | | 14.1±9.6 |
| Exercise habit (%) | | 54.3 |
| Cerebrovascular disease (%) | | 14.2 |
| Ischemic heart disease (%) | | 18.1 |
| Peripheral artery disease (%) | | 11.7 |
| Retinopathy (NDR/SDR/PDR, Post PC, %) ^§^ | | 76.4/10.9/12.7 |
| Nephropathy (stage 1/2/≥3, %) ^¶^ | | 65.4/26.0/8.6 |
| ***Medication*** | |  |
|  | Hypertension (%) | 68.1 |
|  | Dyslipidemia (%) | 56.6 |
| ***Blood markers*** | |  |
|  | Albumin (mg/dL) | 4.2±0.4 |
|  | Creatinine (mg/dL) | 0.9±0.4 |
|  | HbA1c (%) | 7.4±1.3 |
| ***Sarcopenia indices*** | |  |
|  | Sarcopenia (%) | 7.0 |
|  | Skeletal mass index (kg/m^2^) | 7.5±1.2 |
|  | Low skeletal mass index (%) | 13.5 |
|  | Grip strength (kg) | 28.3±9.4 |
|  | Weak grip strength (%) | 21.4 |
|  | Usual gait speed (m/sec) | 1.17±0.26 |
|  | Slow usual gait speed (%) | 25.7 |
|  | Arm muscle quality | 6.5±1.6 |

Values are the mean±standard deviation or frequency. Statistical significance was assessed by analysis of variance or a chi-squared test. Sarcopenia was defined as weak grip strength (<26 kg for men, <18 kg for women) or slow usual gait speed (<1.0 m/sec) and low skeletal mass index (<7.0 kg/m^2^ for men, <5.7 kg/m^2^ for women). Arm muscle quality was calculated by dividing grip strength by arm muscle mass.

BMI: body mass index; NDR: no diabetic retinopathy, SDR: simple diabetic retinopathy, PDR: proliferative diabetic retinopathy, PC: photocoagulation.

Data are available for ^†^ 729, ^‡^ 698, ^§^ 709, and ^¶^ 684 type 2 patients.
