# Supplementary material for: Hyperglycemia in non‐obese patients with type 2 diabetes is associated with low muscle mass: The Multicenter Study for Clarifying Evidence for Sarcopenia in Patients with Diabetes Mellitus
Source: J Diabetes Investig. 2019 Jun 1;10(6):1471–9. doi: 10.1111/jdi.13070 (PMC6825926; doi:10.1111/jdi.13070)
Supplement: Supplementary file 6 — Table S2 | Sex differences in clinical characteristics of the study patients. [file JDI-10-1471-s006.docx]

**Supplementary table 2**. Sex differences in clinical characteristics of the study patients

|  | | Men | Women |
| --- | --- | --- | --- |
|  | | (450) | (296) |
| Age (years) | | 69.9±8.9 | 69.9±9.4 |
| BMI (kg/m^2^) | | 24.5±3.6 | 25. ±4.7 |
| Fat mass (kg) | | 16.4±7.1 | 20.7±9.4 |
| Duration of diabetes (years) ^†^ | | 15.9±10.6 | 15.3±9.5 |
| Duration of treatment of diabetes (years) ^‡^ | | 14.1±9.8 | 14.0±9.2 |
| Exercise habit (%) | | 59.1 | 47.0 |
| Cerebrovascular disease (%) | | 15.6 | 12.2 |
| Ischemic heart disease (%) | | 22.2 | 11.8 |
| Peripheral artery disease (%) | | 13.3 | 9.1 |
| Retinopathy (NDR/SDR/PDR or Post PC, %) ^§^ | | 77.2/11.3/11.5 | 75.3/10.3/14.5 |
| Nephropathy (stage 1/2/≥3, %) ^¶^ | | 63.0/27.1/9.9 | 69.0/24.4/6.6 |
| ***Medication*** | Hypertension (%) | 68.0 | 68.2 |
|  | Dyslipidemia (%) | 51.6 | 64.2 |
| ***Plasma markers*** | Albumin (mg/dL) | 4.3±0.4 | 4.2±0.4 |
|  | Creatinine (mg/dL) | 1.0±0.5 | 0.7±0.2 |
|  | HbA1c (%) | 7.3±1.3 | 7.5±1.4 |
| ***Sarcopenia indices*** | Sarcopenia (%) | 7.1 | 6.8 |
|  | Skeletal mass index (kg/m^2^) | 8.1±1.1 | 6.7±0.9 |
|  | Low skeletal mass index (%) | 14.2 | 12.5 |
|  | Grip strength (kg) | 33.2±8.2 | 20.8±5.3 |
|  | Weak grip strength (%) | 16.7 | 28.7 |
|  | Usual gait speed (m/sec) | 1.18±0.25 | 1.15±0.27 |
|  | Slow usual gait speed (%) | 22.7 | 30.4 |
|  | Arm muscle quality | 6.8±1.6 | 6.1±1.5 |

Values are the mean±standard deviation or frequency.

Sarcopenia was defined as weak grip strength (<26 kg for men, <18 kg for women) or slow usual gait speed (<1.0 m/sec) and low skeletal mass index (<7.0 kg/m^2^ for men, <5.7 kg/m^2^ for women). Arm muscle quality was calculated by dividing grip strength by arm muscle mass.

BMI: body mass index; NDR: no diabetic retinopathy, SDR: simple diabetic retinopathy, PDR: proliferative diabetic retinopathy, PC: photocoagulation.

Data are available for ^†^ 729 (men: 442, women: 287), ^‡^ 698 (men: 420, women: 278), ^§^ 709 (men: 426, women: 283), and ^¶^ 684 (men: 413, women: 271) patients.
