# Supplementary material for: Hyperglycemia in non‐obese patients with type 2 diabetes is associated with low muscle mass: The Multicenter Study for Clarifying Evidence for Sarcopenia in Patients with Diabetes Mellitus
Source: J Diabetes Investig. 2019 Jun 1;10(6):1471–9. doi: 10.1111/jdi.13070 (PMC6825926; doi:10.1111/jdi.13070)
Supplement: Supplementary file 8 — Table S4 | Full results of regression analysis for sarcopenia indices. [file JDI-10-1471-s008.docx]

**Supplementary table 4**. Full results of regression analysis for sarcopenia indices (n=746)

|  | | Low skeletal muscle index | |  | Weak grip strength | |  | Slow gait speed | |
| --- | --- | --- | --- | --- | --- | --- | --- | --- | --- |
|  | | OR (95% CI) | *P* |  | OR (95% CI) | *P* |  | OR (95% CI) | *P* |
| Age (years) | | 1.09 (1.05−1.13) | <0.001 |  | 1.09 (1.06−1.12) | <0.001 |  | 1.06 (1.03−1.08) | <0.001 |
| Sex (men) | | 1.70 (0.95−3.04) | 0.073 |  | 0.48 (0.32−0.72) | <0.001 |  | 0.73 (0.51−1.05) | 0.092 |
| Body mass index (kg/m^2^) | | 0.51 (0.45−0.59) | <0.001 |  | 0.91 (0.87−0.96) | <0.001 |  | 1.05 (1.01−1.10) | 0.024 |
| Exercise habit | | 0.70 (0.41-1.20) | 0.196 |  | 0.47 (0.31-0.69) | <0.001 |  | 0.44 (0.31-0.63) | <0.001 |
| Serum albumin (mg/dl) | | 1.71 (0.80−3.68) | 0.163 |  | 0.24 (0.13−0.43) | <0.001 |  | 0.54 (0.33−0.91) | 0.019 |
| Oral antihyperglycemic drugs (n) | | 1.06 (0.83−1.37) | 0.633 |  | 1.15 (0.97−1.37) | 0.103 |  | 1.17 (1.01−1.36) | 0.042 |
| Insulin therapy | | 1.55 (0.80−3.00) | 0.195 |  | 1.00 (0.61−1.64) | 0.989 |  | 1.29 (0.82−2.02) | 0.264 |
| Cardiovascular diseases | | 1.08 (0.61−1.92) | 0.796 |  | 1.22 (0.79−1.87) | 0.363 |  | 1.64 (1.12−2.39) | 0.011 |
| HbA1c | <6.5% | reference | |  | reference | |  | reference | |
|  | ≥6.5 and <7.0% | 3.29 (1.31−8.23) | 0.011 |  | 1.07 (0.55−2.07) | 0.834 |  | 0.66 (0.37−1.16) | 0.153 |
|  | ≥7.0 and <8.0% | 2.61 (1.09−6.24) | 0.030 |  | 1.46 (0.80−2.67) | 0.221 |  | 1.00 (0.60−1.65) | 0.994 |
|  | ≥8.0% | 5.35 (2.07−13.86) | <0.001 |  | 1.95 (1.00−3.80) | 0.050 |  | 1.16 (0.65−2.06) | 0.612 |

Sarcopenia was defined as weak grip strength (<26 kg for men, <18 kg for women) or slow usual gait speed (<1.0 m/sec) and low skeletal mass index (<7.0 kg/m^2^ for men, <5.7 kg/m^2^ for women). Low arm muscle quality was defined as the lower 20th percentile calculated within sex.

Cardiovascular diseases include ischemic heart diseases, cerebrovascular diseases, and peripheral artery diseases. Renal dysfunction was defined as urinary albumin ≥300 mg/day.
