# Supplementary material for: Hyperglycemia in non‐obese patients with type 2 diabetes is associated with low muscle mass: The Multicenter Study for Clarifying Evidence for Sarcopenia in Patients with Diabetes Mellitus
Source: J Diabetes Investig. 2019 Jun 1;10(6):1471–9. doi: 10.1111/jdi.13070 (PMC6825926; doi:10.1111/jdi.13070)
Supplement: Supplementary file 9 — Table S5 | Clinical characteristics of the Nagahama study population. [file JDI-10-1471-s009.docx]

**Supplementary table 5**. Clinical characteristics of the Nagahama study population

|  | | Total |  | Subpopulation | |
| --- | --- | --- | --- | --- | --- |
|  | |  |  | T2DM | Control |
| N | | 2,067 |  | 212 | 1,855 |
| Age (years) | | 68.2±5.4 |  | 69.6±5.3 | 68.1±5.4 |
| Sex (male, %) | | 34.8 |  | 52.8 | 32.7 |
| Body mass index (kg/m^2^) | | 22.4±3.0 |  | 23.9±3.5 | 22.2±2.9 |
| Cardiovascular disease (%) | | 6.8 |  | 14.2 | 6.0 |
| ***Glycemic traits*** | |  |  |  |  |
|  | Glucose (mg/dl) | 90±15 |  | 114±27 | 88±9 |
|  | HbA1c (%) | 5.7±0.5 |  | 6.6±0.7 | 5.6±0.3 |
|  | Antihyperglycemic medication (%) | 7.1 |  |  |  |
|  | Type 2 diabetes (%) | 10.3 |  |  |  |
| ***Sarcopenia indices*** | |  |  |  |  |
|  | Sarcopenia (%) | 6.1 |  | 6.1 | 6.2 |
|  | Skeletal mass index (kg/m^2^) | 6.6±0.9 |  | 7.1±1.0 | 6.6±0.9 |
|  | Low skeletal mass index (%) | 21.1 |  | 14.6 | 21.8 |
|  | Grip strength (kg) | 27.0±8.5 |  | 28.8±8.9 | 26.8±8.4 |
|  | Weak grip strength (%) | 10.9 |  | 11.8 | 10.8 |
|  | Usual gait speed (m/sec) | 1.39±0.20 |  | 1.34±0.21 | 1.40±0.20 |
|  | Slow usual gait speed (%) | 3.1 |  | 6.1 | 2.8 |

Values are the mean±standard deviation or frequency.

Cardiovascular diseases include symptomatic myocardial infarction and stroke. Type 2 diabetes (T2DM) was defined as glucose ≥126 mg/dl (fasting) or ≥200 mg/dl (nonfasting), HbA1c ≥6.5%, or antihyperglycemic treatment. Sarcopenia was defined as weak hand grip (<26 kg for men, <18 kg for women) or slow usual gait speed (<1.0 m/sec) and low skeletal mass index (<7.0 kg/m^2^ for men, <5.7 kg/m^2^ for women).
