# Supplementary material for: Hyperglycemia in non‐obese patients with type 2 diabetes is associated with low muscle mass: The Multicenter Study for Clarifying Evidence for Sarcopenia in Patients with Diabetes Mellitus
Source: J Diabetes Investig. 2019 Jun 1;10(6):1471–9. doi: 10.1111/jdi.13070 (PMC6825926; doi:10.1111/jdi.13070)
Supplement: Supplementary file 10 — Table S6 | Clinical characteristics of the Septuagenarians, Octogenarians, Nonagenarians Investigation with Centenarians study population. [file JDI-10-1471-s010.docx]

**Supplementary table 6**. Clinical characteristics of the SONIC study population

|  | | Total |  | Subpopulation | |
| --- | --- | --- | --- | --- | --- |
|  | |  |  | SONIC 70 | SONIC 80 |
| N | | 559 |  | 311 | 248 |
| Age (years) | | 80.5±5.2 |  | 75.9±0.9 | 86.2±0.9 |
| Sex (male, %) | | 53.7 |  | 54.0 | 53.2 |
| Body mass index (kg/m^2^) | | 22.6±3.0 |  | 22.8±2.9 | 22.4±3.0 |
| Cardiovascular disease (%) ^†^ | | 27.7 |  | 28.7 | 26.7 |
| ***Glycemic traits*** | |  |  |  |  |
|  | Glucose (mg/dl) | 119±36 |  | 115±32 | 123±40 |
|  | HbA1c (%) | 5.9±0.6 |  | 6.0±0.6 | 5.8±0.6 |
|  | Antihyperglycemic medication (%) ^‡^ | 9.9 |  | 9.8 | 10.1 |
|  | Type 2 diabetes (%) ^‡^ | 16.7 |  | 17.1 | 16.1 |
| ***Sarcopenia indices*** | |  |  |  |  |
|  | Sarcopenia (%) | 15.7 |  | 5.8 | 28.2 |
|  | Skeletal mass index (kg/m^2^) | 6.9±1.1 |  | 7.1±1.1 | 6.6±1.0 |
|  | Low skeletal mass index (%) | 30.6 |  | 19.3 | 44.8 |
|  | Grip strength (kg) | 23.9±7.7 |  | 26.1±8.0 | 21.0±6.3 |
|  | Weak grip strength (%) | 39.9 |  | 25.1 | 58.5 |
|  | Usual gait speed (m/sec) | 1.45±0.33 |  | 1.54±0.31 | 1.34±0.31 |
|  | Slow usual gait speed (%) | 8.6 |  | 3.5 | 14.9 |

Values are the mean ± standard deviation or frequency.

Cardiovascular diseases include symptomatic myocardial infarction and stroke. Type 2 diabetes was defined as ad libitum glucose ≥200 mg/dl, HbA1c ≥6.5%, or antihyperglycemic treatment. Sarcopenia was defined as weak hand grip (<26 kg for men, <18 kg for women) or slow usual gait speed (<1.0 m/sec) and low skeletal mass index (<7.0 kg/m^2^ for men, <5.7 kg/m^2^ for women).

Data are available for ^†^ 505 and ^‡^ 534 participants.
