# Supplementary material for: Hyperglycemia in non‐obese patients with type 2 diabetes is associated with low muscle mass: The Multicenter Study for Clarifying Evidence for Sarcopenia in Patients with Diabetes Mellitus
Source: J Diabetes Investig. 2019 Jun 1;10(6):1471–9. doi: 10.1111/jdi.13070 (PMC6825926; doi:10.1111/jdi.13070)
Supplement: Supplementary file 11 — Data S1 | Supplementary methods. [file JDI-10-1471-s011.docx]

Supplemental materials

**Hyperglycemia in non-obese patients with type 2 diabetes**

**was associated with low muscle mass**

**The MUSCLES-DM study**

**Correspondence to:**

Hiromi Rakugi

Department of Geriatric and General Medicine,

Osaka University Graduate School of Medicine

Yamadaoka 2-2 B6, Suita, 565-0871 Japan

Tel.: +81-6-6879-3852

Fax: +81-6-6879-3859

E-mail: rakugi@geriat.med.osaka-u.ac.jp

**Supplementary methods**

***The Nagahama study***

We analyzed the second visit dataset (follow-up measurement) describing participants in the Nagahama Prospective Cohort for Comprehensive Human Bioscience (the Nagahama study) [*J Hypertens*. 2018; 36:587-593; *J Hypertens*. 2018; *36*:1147-1153.]. The participants were recruited between 2008 and 2010 (baseline measurement) from the general population of Nagahama City, a rural city of approximately 125,000 people, located in central Japan. The eligible participants were community residents aged 30–74 years, living independently without any physical impairment or dysfunction. From a total of 9,764 individuals in the baseline population, 8,289 participated in the follow-up survey between 2013 and 2015, performed five years after the baseline evaluation. By further recruiting 1,561 participants meeting the inclusion criteria, the second visit dataset of this cohort comprised 9,850 participants.

Among them, a total of 2,121 individuals participated in a physical performance test during the second visit, which was an optional examination for participants aged 60 years or older (n=5,018). After excluding 54 individuals for whom any of the physical performance data required for diagnosis of sarcopenia were missing or had incomplete measurements, a total of 2,067 participants were ultimately included in the analysis. Other clinical parameters used in this study were obtained at the follow-up measurement. T2DM was defined as glucose ≥126 mg/dl (fasting) or ≥200 mg/dl (nonfasting), HbA1c ≥6.5%, or antihyperglycemic treatment.

All study procedures were approved by the Ethics Committee of the Kyoto University Graduate School of Medicine and by the Nagahama Municipal Review Board. Written informed consent was obtained from all participants.

## The SONIC study

A dataset of the Septuagenarians, Octogenarians, Nonagenarians Investigation with Centenarians (SONIC) study [*J Dent*. 2014; 42:556-564; *Hypertens Res*. 2016; 39:557-563.] was used for the analysis. The SONIC study is a longitudinal multidisciplinary study aiming to investigate factors for noncommunicable diseases and longevity in older Japanese. The study participants were recruited in two regions of eastern and western Japan (Tokyo metropolitan and Hyogo prefecture, respectively) from randomly selected community residents aged 69−71 (SONIC 70) or 79−81 (SONIC 80) during the baseline investigation (July 2010 − March 2011). This study analyzed a dataset obtained at the second follow-up measurement at the Hyogo study site performed during 2016–2017. Among the participants, individuals whose clinical and physical performance data required for the analysis were available were ultimately included in the analysis [SONIC 70: n=311 (aged 75–77 years), SONIC 80: n=248 (aged 85−87 years)]. All clinical and physical data were obtained at the follow-up measurement. T2DM was defined as ad libitum glucose ≥200mg/dl, HbA1c ≥ 6.5%, or antihyperglycemic treatment.

The procedures of the SONIC study were approved by the Institutional Review Board at Osaka University Graduate School of Medicine. Written informed consent was obtained from all the participants.

***Measurement of grip strength and usual gait speed***

We measured grip strength and gait speed by slightly different methods from that adopted in the MUSCLES-DM study.

The grip strength of the dominant hand (SONIC study) or both hands (Nagahama study) was measured using a standard digital grip dynamometer [Grip-D, Takei Scientific Instrument Co., Ltd., Japan, and YD, TTM, Tokyo, Japan (Nagahama study), or Model YD-100, Yagami Ltd., Tokyo, Japan (SONIC study)]. Measurements were taken twice in a sitting position with arm position horizontal (SONIC study) or vertical (Nagahama study) to the ground. The mean values of all the measurements were used for the analysis.

The usual gait speed measurements in the SONIC study used a 2.44 m walkway without an approach way, and the elapsed time was measured manually. In the Nagahama study, photoelectric sensors placed 1, 2, 4, 8, and 10 m from the starting line on a 12 m walkway (Brower Timing Systems, Co., Ltd., UT, USA) were used for the elapsed time measurements. The gait speed was calculated by the elapsed time difference between the points at 1 and 8 m. Measurements were taken twice, and the average value was used for the analysis. The gait speeds measured by the different method were adjusted using equations (Supplementary Figure 1), which were calculated using multipoint measured gait speed data in the Nagahama study, to avoid potential misclassification.

**FIGURE LEGENDS**

**Supplementary figure 1**. Different associations between walkway distance and usual gait speed by the point of gait speed calculation

Elapsed times for 1, 2, 4, 8, and 10 m walks from the starting line were measured using a photoelectric sensor (Brower Timing Systems, Co., Ltd., UT, USA). The gait speed was calculated using the following two ranges: 0 to 10 m (without an approach way) and 1 to 10 m (1 m approach way). The second- and first-order regression equations were calculated using the available data, respectively. The number of study participants in each measurement point is shown in the parentheses.

**Supplementary figure 2**. Association between HbA1c levels and sarcopenia in a general population (the Nagahama study)

Frequency of sarcopenia by body mass index and plasma HbA1c levels. The numbers of participants in each body mass index subgroup are shown in the parentheses.

**Supplementary figure 3**. Association between HbA1c levels and sarcopenia in an older adult population (the SONIC study)

Frequency of sarcopenia by body mass index and plasma HbA1c levels. The numbers of participants in each body mass index subgroup are shown in the parentheses.

**Supplementary figure 4**. Adjusted odds ratio for sarcopenia in non-obese individuals (the SONIC study)

Participants whose body mass index were greater than 25 kg/m^2^ were excluded from the analysis. The odds ratio adjusted for age, sex, and body mass index was calculated using the intermediate HbA1c subgroup as a reference.
